# Large-Scale Monitoring of Resistance to Coumaphos, Amitraz, and Pyrethroids in *Varroa destructor*

**DOI:** 10.3390/insects12010027

**Published:** 2021-01-04

**Authors:** Carmen Sara Hernández-Rodríguez, Óscar Marín, Fernando Calatayud, María José Mahiques, Ana Mompó, Inmaculada Segura, Enrique Simó, Joel González-Cabrera

**Affiliations:** 1 Instituto Universitario de Biotecnología y Biomedicina (BIOTECMED), Universitat de València, Dr. Moliner 50, 46100 Burjassot, Spain; sara.hernandez@uv.es (C.S.H.-R.); oscar.marin@uv.es (Ó.M.); 2 Agrupación de Defensa Sanitaria Apícola APIADS, Calle Raval 75B, 46193 Montroi, Spain; fernancaltor@gmail.com (F.C.); enrisimo@gmail.com (E.S.); 3 Agrupación de Defensa Sanitaria Apícola APICAL y APIVAL, C/Sants de la Pedra 75, 03830 Muro de Alcoy, Spain; mahiques.mj@gmail.com (M.J.M.); anamompo1@gmail.com (A.M.); rusca00@gmail.com (I.S.)

**Keywords:** acaricides, honey bees, bioassay, TaqMan, genotyping, acaricide resistance

## Abstract

**Simple Summary:**

*Varroa destructor*, a parasitic mite of *Apis mellifera*, is causing severe damages to honey bee colonies worldwide. There are very few acaricides available to manage the parasite, and so the evolution of the mite’s resistance to acaricides poses a serious threat to controlling the mite. Using a combined approach that includes bioassays and genotyping, we estimated the expected efficacy of the treatments with acaricide products based on coumaphos, amitraz, and pyrethroids in apiaries from one of the most important beekeeping regions in Spain. This information was shared with the beekeeping community so that they can take informed and scientific-based decisions in the most convenient way to manage the parasite.

**Abstract:**

*Varroa destructor* is an ectoparasitic mite causing devastating damages to honey bee colonies around the world. Its impact is considered a major factor contributing to the significant seasonal losses of colonies recorded every year. Beekeepers usually rely on a reduced set of acaricides to manage the parasite, usually the pyrethroids tau-fluvalinate or flumethrin, the organophosphate coumaphos, and the formamidine amitraz. However, the evolution of resistance in the mite populations is leading to an unsustainable scenario with almost no alternatives to reach an adequate control of the mite. Here, we present the results from the first large-scale and extensive monitoring of the susceptibility to acaricides in the Comunitat Valenciana, one of the most prominent apicultural regions in Spain. Our ultimate goal is to provide beekeepers with timely information to help them decide what would be the best alternative for a long-term control of the mites in their apiaries. Our data show that there is a significant variation in the expected efficacy of coumaphos and pyrethroids across the region, indicating the presence of a different ratio of resistant individuals to these acaricides in each population. On the other hand, the expected efficacy of amitraz was more consistent, though slightly below the expected efficacy according to the label.

## 1. Introduction

The ectoparasitic mite *Varroa destructor* is considered a major pest of the Western honey bee (*Apis mellifera* L.) [1]. This mite feeds mostly on the fat body of immature and adult bees and vectors numerous lethal viruses, compromising the natural honey bee defenses [2,3]. These severe damages make *V. destructor* one of the main factors contributing to the many seasonal losses of honey bee colonies around the world [4,5].

*Varroa destructor* shifted its host from the Eastern honey bee (*Apis cerana* F.) to the Western honey bee in the late 1950s in Asia, but at present it is widely distributed throughout the world [6]. In Spain, *V. destructor* was first detected in 1985, and currently it can be found all over the country [7,8].

*Varroa destructor* reproduces throughout the spring and summer, and so the population is larger in autumn. Thus, treatments to control the mite are usually applied in that season to increase the possibility of overwintering success [9]. In this country, as in many others, it is mandatory to apply at least one acaricide treatment per year to manage the parasite (Royal Decree RD608/2006). However, beekeepers usually perform at least another treatment in the summer in case they detect mites in their colonies. The acaricides authorized to control *V. destructor* in Spain include “hard acaricides” (based on pyrethroids such as tau-fluvalinate and flumethrin, the formamidine amitraz, and the organophosphate coumaphos), together with “soft acaricides” (mostly based on formic or oxalic acid or the essential oil thymol) ([10]; www.aemps.gob.es/). Integrated pest management (IPM) strategies encourage the combined use of both types of acaricides and other beekeeping practices to reach better long-term control of the mite, but beekeepers have relied mainly on hard acaricides because they are faster and usually more effective [1].

The intensive use of pyrethroids to control Varroa for decades resulted in the evolution of the mite’s resistance to these acaricides in apiaries from several countries [11,12,13,14,15]. Since the emergence of resistance to pyrethroids, beekeepers switched to coumaphos as the best alternative to control the parasite, but the intensive treatment regime with this compound resulted in the evolution of resistance in many locations as well [16,17,18]. In this scenario, the alternatives to control *V. destructor* have been drastically reduced to a treatment with amitraz and soft acaricides. Currently, the extensive use of amitraz exerts an intense selection pressure over populations, threatening them with the evolution of resistance to this compound. Indeed, a reduction in the efficacy of amitraz for Varroa control, which may be associated with the evolution of its resistance, has already been reported elsewhere [19,20,21,22].

The mechanism of resistance to pyrethroids in *V. destructor* is well known. It is associated with mutations at the residue L925 of the major target site for pyrethroids—the voltage-gated sodium channel (VGSC) [12,23,24,25]. To detect these amino acid substitutions, TaqMan allelic discrimination assays have been developed [12,23,24]. This is a high throughput diagnostics technique capable of detecting mutations in individual mites. On the other hand, the molecular mechanisms causing the resistance to coumaphos and amitraz in *V. destructor* are still unknown, and so the reduction in the efficacy to control *V. destructor* in apiaries using treatments based on these two acaricides can only be confirmed by bioassays with a direct exposition of mites to the acaricidal products [16,17,18].

The European Union (EU) is the world’s second largest honey producer after China. Spain is the country with the highest number of hives in Europe (more than three million hives) and the second largest producer of honey in the EU, with almost 30,000 tons per year (https://ec.europa.eu/info/food-farming-fisheries/animals-and-animal-products/animal-products/honey_en). The Comunitat Valenciana is a Spanish extensive region of 23,255 km^2^ comprising three provinces with an important professionalized beekeeping sector. It has 358,327 hives in 2459 beekeeping operations, being the region with the second highest honey production in Spain. Almost all the hives (98%) are mobile, carrying out migratory beekeeping throughout the year (https://www.mapa.gob.es/es/ganaderia/temas/produccion-y-mercados-ganaderos/indicadoreseconomicossectordelamiel2018comentarios_tcm30-419675.pdf).

Despite the treatments to manage the mite, Varroa parasitism is far from being controlled, and it is a persistent problem in all honey-producing countries [1,26]. It seems plausible that the continuous presence of varroosis in beehives around the world is related to the resistance to acaricidal products. In Spain, this correlation has not been confirmed yet since there is no program to track the efficacy of the treatments or the evolution of resistance in Spanish apiaries. The lack of knowledge of the incidence and prevalence of varroosis in all the territories of this country led us to plan a systematic study in the Comunitat Valenciana region. The goal of this study is to determine the efficacy of the three groups of hard acaricides in *V. destructor* populations from apiaries located throughout the three provinces of the region. This study is coordinated with the Department of Agriculture, Environment, Climate Change and Rural Development of the regional government (Generalitat Valenciana, www.gva.es) and Sanitary Defense Groups (abbreviated as ADS in Spanish) of the beekeeping sector. Our aim is to provide beekeepers with information about the impact of varroosis in their colonies and to estimate the expected efficacy for each apiary of acaricide treatments based on pyrethroids, coumaphos, and amitraz.

## 2. Materials and Methods

### 2.1. Mites

*Varroa destructor* females were collected from apiaries located in the three provinces of the Comunitat Valenciana region (Spain): Castellón, Valencia, and Alicante. The collection of mites for this study was carried out in two consecutive annual beekeeping seasons: In the first period, 90 apiaries were sampled from April to July 2018, and in the second period, 89 different apiaries were sampled from November 2018 to July 2019 (Table 1, Tables S1 and S2). At least two combs with capped brood were collected from one or two colonies per apiary, boxed in polystyrene containers and shipped to the laboratory at the University of Valencia by express courier. The combs arrived in less than 24 h after collection to ensure optimal mite conditions before bioassays. Beekeepers and veterinaries from nine Sanitary Defense Groups (ADS) participated in the collection and shipments of the combs (Table 1). For this study, we defined the sample as the mites collected from each apiary to carry out the experiments.

### 2.2. Bioassays with Acaricides

**Figure 1:**
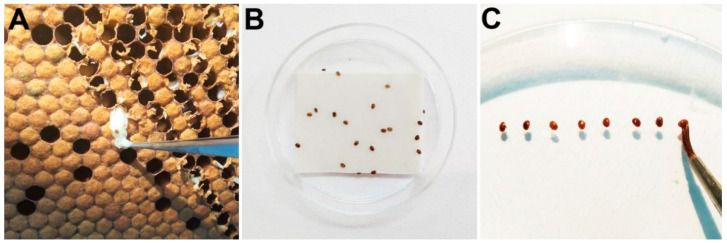
Bioassays with *Varroa destructor*. (**A**) Parasitized bee pupae extracted from the brood cells. (**B**) Female mites laid on top of the acaricide strip inside a Petri dish. (**C**) Mortality was evaluated by assessing the movement of mites after probing them with a fine paint brush.

Bioassays were conducted as in Higes et al. (2020) [26] using strips of Checkmite+ (coumaphos a.i., Bayer, Germany), Apitraz (amitraz a.i., Laboratorios Calier, S.A., Spain), Amicel Varroa (amitraz a.i., Maymó S.L., Spain), and Apivar (amitraz a.i., VétoPharma, France). Briefly, parasitized bee pupae were extracted from the brood cells using a pair of soft tweezers (Figure 1A). The female mites were collected with a soft paint brush and deposited onto a wet filter paper. A piece of approximately 4 cm long of each acaricide strip was placed into a 5.5 cm Petri dish. Amicel Varroa strips were prepared following the manufacturer instructions. Each strip piece maintained its original width (2.5 cm for Checkmite+; 4.0 cm for Apitraz and Apivar; and 4.6 cm for Amicel Varroa). Given the different amounts of active ingredient impregnated in the strips of each product and considering the surface of both sides of the strips, the actual concentration was 13.6, 2.1, 0.8, and 3.1 mg/cm^2^ for Checkmite+, Apitraz, Amicel Varroa, and Apivar, respectively. The mites (15 mites per replicate, 1–3 replicates for each acaricide product) were laid on top of the strip, and their movements were monitored to ensure that they remain on top of the strip for at least 5 min (Figure 1B). The dish was sealed with Parafilm holed with an entomological needle to allow for aeration. Mites placed onto the acaricide strip were incubated for 1 h at 34 °C, 90% RH in a wet incubator. After 1 h, the strip was removed, and the dish with the mites was incubated for 3 more hours at 34 °C, 90% RH. Control mites were treated the same way, but the mites were placed on top of a piece of filter paper (Whatman^®^ grade 1, 4 × 2.5 cm) instead of acaricide strips. After the incubation time was completed, mortality was evaluated by assessing the movement of mites after probing them with a fine paint brush (Figure 1C). The expected efficacy of each acaricide was estimated using the mortality values obtained in the bioassays after correction with the mortality in the controls using the Schneider–Orelli’s formula [27]. Mortality in the controls was always below 10%.

To conduct a complete set of experiments, at least 180 live mites per apiary were needed, but the number of mites collected from the combs received in the laboratory did not reach that limit in some cases. Giving the nature of this project, even in those cases with a lower number of mites, we decided to conduct as many experiments as possible, resulting in some bioassays with only two or even one replicate (Tables S1 and S2).

### 2.3. TaqMan Assays

The genotyping of mites for detecting susceptible and pyrethroid-resistant alleles in a *V. destructor* VGSC was carried out using a TaqMan-based allelic discrimination assay as described by González-Cabrera et al. (2013) [23]. Genomic DNA was extracted from individual adult mites by an alkaline hydrolysis method and stored at −20 °C until used. Briefly, reactions mixtures contained 1.5 µL of genomic DNA, 7.5 µL of TaqMan’s Fast Advanced Master Mix (Thermo Fisher Scientific, Waltham, MA, USA), 0.9 µM of each primer, and 0.2 µM of each fluorescent-labelled probe in a total reaction volume of 15 µL. Assays were run on a StepOne Plus Real-Time PCR system (Thermo Fisher Scientific, Waltham, MA, USA) using the following temperature cycling conditions: 10 min at 95 °C followed by 40 cycles of 95 °C for 15 s and 60 °C for 45 s. The increase in VIC and 6FAM™ fluorescence was monitored in real time by acquiring each cycle on the yellow channel (530 nm excitation and 555 nm emission) and the green channel (470 nm excitation and 510 emission) of the StepOne Plus, respectively. Forty mites were genotyped per apiary. Duplicated control samples corresponding to the homozygous for the resistant allele (RR), homozygous for the susceptible allele (SS), heterozygotes (SR) and negative controls (distilled water) were included in each assay. Since resistance to pyrethroids associated to amino acid substitutions in the VGSC is inherited as a recessive trait [23,28], mites carrying the mutant allele in homozygosis (RR) are considered as pyrethroid-resistant, whereas mites carrying the wild-type allele in homozygosis (SS) and the heterozygotes (SR) are considered as pyrethroid-susceptible mites. In this case, the expected efficacy was estimated as the percentage of susceptible mites in the pool (sum of mites with SS and SR genotypes).

### 2.4. Statistical Analysis

The unpaired and nonparametric Mann–Whitney test was used to compare the expected efficacies between Apitraz and Amicel Varroa products in 2018, and between Apitraz and Apivar products in 2019. One-way ANOVA on rank tests (Kruskal-Wallis tests) were used to compare the expected efficacies of each acaricide among provinces. The *p* values lower than 0.05 were considered as statistically significant. The calculations were conducted using GraphPad Prism version 7.0 (GraphPad Software, Inc., San Diego, CA, USA).

## 3. Results

The samples (mites collected from capped brood) used in this study were collected from apiaries located across the three provinces of the Comunitat Valenciana region (Spain). The level of parasitism that was required for conducting the bioassays was found in 58% and 81% of the apiaries sampled in 2018 and 2019, respectively. On the other hand, since it is possible to carry out TaqMan assays using a smaller number of mites collected either dead or alive, more analyses were carried out with this technique than with bioassays, resulting in 70% and 94% of the apiaries tested in the 2018 and 2019 seasons, respectively.

**Figure 2:**
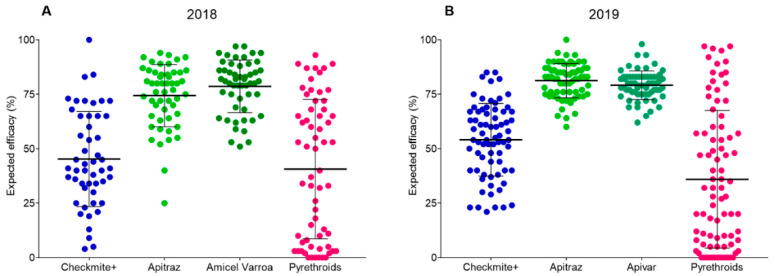
Expected efficacy of commercial acaricides against *Varroa destructor* in the 2018 season (**A**) and 2019 season (**B**). Each dot corresponds to the sample from an apiary. Long horizontal bars represent the mean values and error bars indicate the standard deviation (SD). For Checkmite+, Apitraz, Amicel, and Apivar, the expected efficacy corresponds to mortality recorded in the bioassays. The expected efficacy of pyrethroids-based acaricides was estimated using the frequency of pyrethroid-resistant (RR) and susceptible mites (SS + SR) after genotyping individual mites for the presence of different alleles of the mutation L925V at the *V. destructor* VGSC.

Results from bioassays conducted with Checkmite+ strips (coumaphos a.i.) showed some variability among samples, with a mean mortality of 50% (±21 SD) in 2018 (Figure 2A) and 54% (±17 SD) in 2019 (Figure 2B). Overall, 75% of the 122 apiaries tested in both seasons showed an expected efficacy below 66%, indicating that this product was less effective than expected according to the label.

Three commercial acaricides based on amitraz were tested: Apitraz, Amicel Varroa, and Apivar. Results from bioassays carried out with Apitraz in 2018 showed a mean mortality of 74% (±14 SD) (Figure 2A). In that season, bioassays with Amicel Varroa showed a mean mortality of 79% (±12 SD) (Figure 2A). The statistical analysis shows that these values were not significantly different (Mann–Whitney U = 1133, *p* > 0.05). In 2019, the mean mortality with Apitraz and Apivar was 81% (±8 SD) and 79% (±7 SD), respectively (Figure 2B), again with no significant differences between them (Mann–Whitney U = 1807, *p* > 0.05). Therefore, according to our data, the mortality of the three amitraz-based acaricides tested was found to be similar across the study.

The expected efficacy of pyrethroids based acaricides against *V. destructor* was estimated using a TaqMan^®^ genotyping assay. The frequency of pyrethroid-resistant and susceptible mites was determined for each sample after genotyping 40 individual mites for the presence of different alleles of the mutation L925V at the *V. destructor* VGSC. When TaqMan assays were conducted, a wide range of allele frequency patterns was found in the apiaries evaluated in both, the 2018 and 2019 seasons. The estimated mean efficacy of pyrethroids was 41% (±32 SD) in samples from 2018 (Figure 2A), and 36% (±32 SD) in samples from 2019 (Figure 2B). The standard deviation of these means corroborated the high dispersion of pyrethroid-expected efficacies throughout the region, ranging from zero (in apiaries with all mites resistant to pyrethroids) to 97% in some apiaries with almost all mites susceptible to the acaricide.

**Figure 3:**
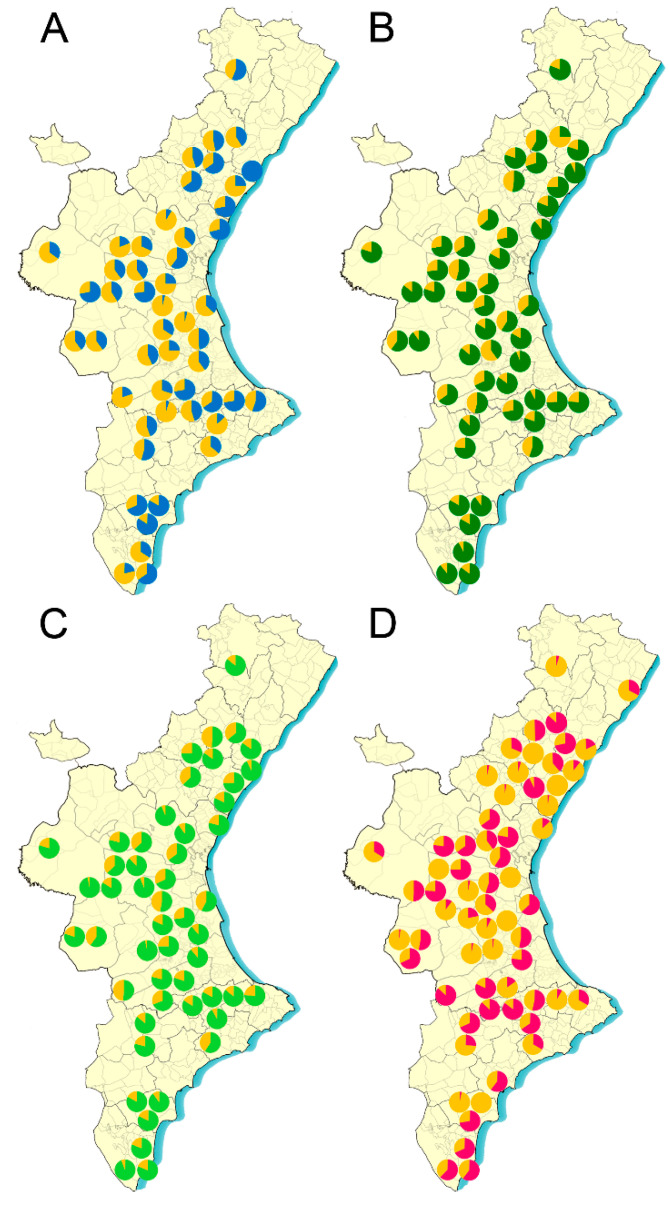
Sampling locations and expected efficacy (expressed as percentage) of acaricides against *Varroa destructor* in 2018 season. (**A**) Checkmite+ (efficacy in blue); (**B**) Apitraz (efficacy in dark green); (**C**) Amicel Varroa (efficacy in light green); (**D**) Pyrethroid-based acaricides (efficacy in pink).

**Figure 4:**
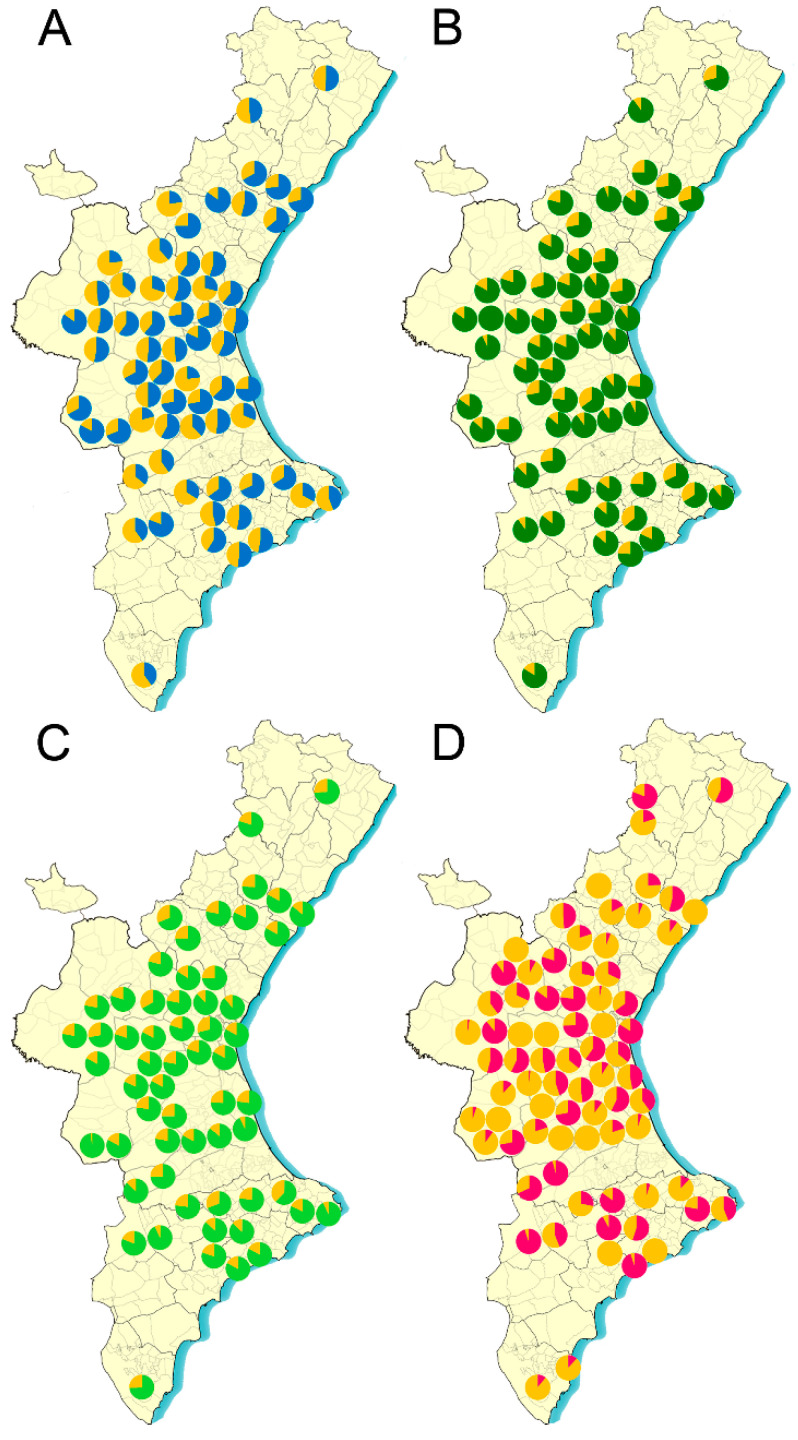
Sampling locations and expected efficacy (expressed as percentage) of acaricides against *Varroa destructor* in the 2019 season. (**A**) Checkmite+ (efficacy in blue); (**B**) Apitraz (efficacy in dark green); (**C**) Apivar (efficacy in light green); (**D**) Pyrethroid-based acaricides (efficacy in pink).

The efficacy of the different acaricides in each apiary was weighed according to its geographic location (Figure 3 and Figure 4) throughout the region and no geographic dependent pattern was found (Figure S1) (Kruskal Wallis test, *p* > 0.05).

**Figure 5:**
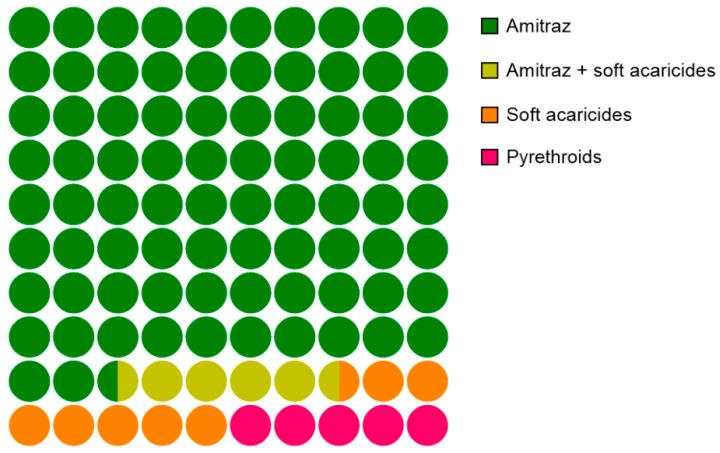
Last acaricide treatment carried out (%) in the apiaries that provided combs for this study in the 2018 and 2019 seasons.

Along with the brood combs, information about treatment history was collected from each apiary. These data show that most of the beekeeping operations used amitraz-based acaricides (88% of the treatments), while the use of other treatment regimens, such as those based on pyrethroids and soft acaricides, was much lower, representing 5% and 7% of the total treatments, respectively (Figure 5).

## 4. Discussion

The information generated in this study is the result of the first comprehensive and large-scale monitoring study describing the situation of the resistance to acaricides in populations of *V. destructor*. The analyzed apiaries belong to the Comunitat Valenciana, one of the most prominent apicultural regions in Spain. Moreover, since migratory beekeeping is a common practice among beekeepers, it is possible to hypothesize that the current situation in this region may resemble that of the rest of the country.

The huge number of apiaries in Spain requires an adequate management of the active ingredients for *V. destructor* control so that the most appropriate approach for each operation is used, depending on the susceptibility of the mite population to each acaricide. However, there is no official program in this country to record the efficacy of treatments, nor the monitoring of possible outbreaks of resistance to these treatments. In a recent study, bioassays with Checkmite+, Apivar, and Apistan were conducted with samples collected in seven Spanish locations [26]; this provided a general idea of the acaricide efficacy in these apiaries. Aiming to perform a more thorough study, we carried out analyses in two consecutive years with a significantly higher number of samples, almost completely covering the area dedicated to beekeeping in this region. To obtain data from a large number of apiaries, we coordinated with the government of the Comunitat Valenciana region (Generalitat Valenciana) and nine sanitary defense groups (ADS) of the beekeeping sector. As a consortium, we started a program to evaluate the efficacy of the authorized active ingredients to control *V. destructor* in apiaries of this Spanish region. The study was carried out during the 2018 and 2019 beekeeping seasons, where the three groups of hard acaricides authorized in Spain (i.e., coumaphos, amitraz, and pyrethroids) were tested.

Coumaphos has been widely used for many years as an active ingredient in Checkmite + commercial strips. In Spain, a reduction of this product efficacy to control *V. destructor* parasitism was detected a few years ago, with mean efficacies of about 70% to 80% [29,30]. Actually, the product’s distributor in Spain, Bayer Hispania, S.L. (Animal Health), issued a statement informing beekeepers of a possible lack of Varroa sensitivity to coumaphos based on the preliminary results obtained in a study with this acaricide in three areas of Central and Northern Spain (https://www.aeapicultores.org/wp-content/uploads/2017/03/Comunicado_Bayer_Apicultores.pdf). After this communication from Bayer, the use of this acaricide declined dramatically, and even the Spanish Association of Veterinary Specialists in Health and Bee Production (AVESPA, Spanish acronym) demanded the withdrawal of the product from the market and discouraged its use among beekeepers (http://www.colvet.es/node/2663). Tracking the efficacy of Checkmite+ strips with mites from the apiaries of this study confirmed the reduction of its efficacy to control Varroa. According to our data, the mortality observed with this acaricide varied considerably from one apiary to another (Figure 3 and Figure 4; Tables S1 and S2), although in all cases the expected efficacy would be lower than that indicated by the manufacturer. The significant variation in the mortality registered for coumaphos in the bioassays throughout the study seems to indicate that the reduced efficacy is due to the presence of mites resistant to this acaricide in the colonies. Actually, our previous study with mites that were sampled in different Spanish regions also found that resistance to coumaphos has evolved in this country [26]. *Varroa destructor* populations resistant to coumaphos were also previously reported in America [16,17,18], where further analysis showed that at least in some cases, the resistance to coumaphos is not reversible after stopping the treatments with this active ingredient [31]. Our results support this hypothesis since the apiaries in this study had not been treated with coumaphos for several years, and still part of the populations remained insensitive to this compound. The accumulation and persistence of coumaphos residues in beeswax over a long time could favor a constant selection pressure in the colonies, in turn reducing the possibility of reversing the resistance [32]. However, to rule out this hypothesis, it may be necessary to identify the molecular mechanism of the resistance, which may also be of help to design molecular tools to identify coumaphos-resistant individuals in the populations.

On the other hand, the mechanism of *V. destructor* resistance to pyrethroids is well described, and for this reason a TaqMan allelic discrimination assay was used to identify the resistant mites carrying a mutation in the 925 position of the VGSC as described by González-Cabrera et al. (2013) [23]. We used the proportion of susceptible and pyrethroid-resistant individuals in each apiary to estimate the efficacy that a pyrethroid treatment would have. The results also show great diversity in the proportion of resistant mites among apiaries. Remarkably, bees on many operations were parasitized only by pyrethroid-resistant mites, but there were also operations where most of the mites were susceptible (Figure 3 and Figure 4; Tables S1 and S2). The long track record of treatments only with pyrethroid-based acaricides and their accumulation in beeswax [32] is a likely explanation for the evolution of resistance. Although it has been suggested several times that the mutations associated with the resistance to pyrethroids in Varroa cause a reduced fitness in the mites [12,24,33], it seems that this is not enough to completely remove the resistant mites. The Varroa populations may contain a remnant of resistant individuals that would be quickly selected as soon as the treatment with pyrethroids are reinstated. This observation again confirms the idea that although it is advisable to use pyrethroids to control Varroa in an integrated pest management context when the frequency of resistant individuals is sufficiently low, it is very important to avoid applying continuous treatments with pyrethroid-based acaricides.

In the case of both coumaphos and pyrethroids, no geographical distribution associated with the resistance was observed. On the contrary, the estimated efficacy for these treatments is randomly distributed. This might be surprising at first sight, but it can be explained by the idiosyncrasy of beekeeping in this region, where beekeepers mainly kept colonies that are seasonally moved across the country. The movement of colonies to different areas most certainly involves a transfer of parasitized bees from the migrant colonies to those already in the transient settlement, and vice versa. This can justify the fact that each apiary has a specific pattern of acaricide efficacy depending on both the treatments applied and the places they have traveled.

Apitraz, Apivar, and Amicel Varroa, i.e., commercial products containing amitraz as an active ingredient, are widely used in Spain. The assays conducted showed that the expected efficacy in the field would be very similar for the three of them, and it would also be the highest amongst the different active ingredients tested in this study (Figure 3 and Figure 4; Tables S1 and S2). The low variation recorded after assaying amitraz in multiple apiaries is an indication of its consistency as acaricide across the country. However, given that our data indicate that the expected efficacy would be below 90% in most cases, it is clear that the products are performing below the expected efficacy according to the label. These data are in agreement with the actual efficacy of amitraz-based product recorded by beekeepers in the field, with values ranging from 60% to 96% [29]. Hence, it is possible to anticipate an evolution of resistance to amitraz in the coming seasons unless there is a significant change in the management strategies, which are currently based on the intensive use of mainly amitraz many times per year. Repeated use of a single acaricide exerts an enormous selection pressure on the mites. If resistance to amitraz evolves, it will be much more difficult to control the parasite, since the other commercial products will not be effective in all apiaries. The recommendations to delay the evolution of resistance encourages the rotation of products with a different mode of action (IRAC; https://www.irac-online.org/). Therefore, a more rational use of current acaricides would be desirable, where the application of different active ingredients is alternated in consecutive seasons. To decide whether a given treatment would be successful in each apiary, it is crucial to monitor the efficacy of the acaricidal compounds in each of the treatments. In this way, the effective products could be rotated, thereby avoiding selection pressures with treatments that can lead to an increase in the frequency of resistant mites.

The transfer from academia to the field is one of the priorities of this work. The final objective is to provide the beekeeping sector with the information obtained in this study. The results from this project were disclosed in informative talks to groups of beekeepers. Moreover, tailored reports with the expected efficacy of acaricide treatments in each apiary were sent to the professionals in charge of the different ADS for them to discuss with the relevant beekeeper the best management approach for controlling the mites in their apiaries, while also considering the previous history of treatments.

Initiatives such as the Honey Bee Health Coalition (https://honeybeehealthcoalition.org/) and the Bee-Informed Partnership (https://beeinformed.org/) in the US (coalitions of researchers, advisors, and stakeholders from various sides of the honey bee related industry) are good examples of associations that encourage the flow of information among the different actors in the beekeeping world. The rational use of pesticides to manage *V. destructor* is a joint responsibility of public institutions, industry, academia, and the beekeeping sector. It is only by acting in a coordinated manner that the efficacy of treatments may be prolonged and the evolution of resistances that threaten the viability of apiculture may be delayed.

## 5. Conclusions

We conducted bioassays and genotyped mites collected in a vast region of the country to estimate the expected efficacy of the treatments with the main acaricides authorized by the Spanish regulator. Our data showed a significant variation in the expected efficacies of all acaricides tested with no particular geographic pattern associated. The highest variation was estimated for coumaphos and pyrethroids, with many apiaries showing very low expected efficacies. This confirmed previous observations indicating that resistance has evolved in these mite populations. However, our data also evidenced that there are apiaries where the efficacy of these compounds is expected to be high. This information together with the still higher and more consistent efficacy of amitraz can be used to design IPM strategies to reach a long-term control of the parasite.

## Figures and Tables

**Table 1:** Sanitary Defense Groups (ADS) that supplied combs for this study in 2018 and 2019 seasons.

Sanitary Defence Groups (ADS)	Province	No. Apiaries Sampled in 2018	No. Apiaries Sampled in 2019
ADSAV	Valencia	6	3
AIXAM	Castellón	5	4
ALAPI	Alicante	2	1
APAC	Castellón	17	13
APIADS	Valencia	23	27
APICAL	Alicante	15	12
APIVAL	Valencia	9	13
CASAPI	Castellón	5	4
PROAPI	Valencia	8	12
TOTAL		90	89

## Data Availability

Data is contained within this article and the supplementary material (Figure S1: Expected efficacy of acaricides in apiaries providing samples from the three provinces of the Comunitat Valenciana region (CS, Castellón; V, Valencia; A, Alicante). Table S1: Sample locations and expected efficacy of acaricides recorded in assays from the 2018 season. Table S2: Sample locations and expected efficacy of acaricides recorded in assays from the 2019 season).

## Supplementary Materials

The following are available online at https://www.mdpi.com/2075-4450/12/1/27/s1, Figure S1: Expected efficacy of acaricides in apiaries providing samples from the three provinces of the Comunitat Valenciana region (CS, Castellón; V, Valencia; A, Alicante). Table S1: Sample locations and expected efficacy of acaricides recorded in assays from the 2018 season. Table S2: Sample locations and expected efficacy of acaricides recorded in assays from the 2019 season.
