# Supplementary material for: Large-Scale Monitoring of Resistance to Coumaphos, Amitraz, and Pyrethroids in Varroa destructor
Source: Insects. 2021 Jan 4;12(1):27. doi: 10.3390/insects12010027 (PMC7824307; doi:10.3390/insects12010027)
Supplement: Supplementary file 1 [file insects-12-00027-s001.pdf]

Table S1. Sample locations and expected efficacy of acaricides recorded in assays from the 2018 season.

Apiary	Location	Expected Efficacy [1] (%)						
		Pyrethroids [2]	Checkmite+	SEM [3]	Apitraz	SEM	Amicel	SEM
18_ADSAV6	Monserrat	3	73	ND [4]	77	ND	94	ND
18_AIXAM1	Altura	3	ND		ND		ND	
18_AIXAM2	Castelló de la Plana	18	69	5	56	5	80	6
18_AIXAM4	Les Alqueries	11	100	ND	93	ND	93	ND
18_ALAPI3	Elx	0	83	5	80	2	90	4
18_APAC01	Betxí	93	ND		ND		ND	
18_APAC02	Onda	0	64	11	72	7	87	4
18_APAC05	Onda	32	46	4	81	4	75	2
18_APAC10	Vila-real	89	ND		ND		ND	
18_APAC11	Sant Joan de Moró	50	47	8	58	2	51	3
18_APAC12	Costur	87	41	12	25	10	64	8
18_APAC13	La Vall d'Uixó	3	72	4	81	7	82	4
18_APAC14	Sta. Magdalena de Pulpis	33	ND		ND		ND	
18_APAC16	Borriol	72	ND		80	6	85	ND
18_APAC17	Ares del Maestre	5	56	12	81	6	86	6
18_APIADS0	Caudete	34	34	2	78	4	81	11
18_APIADS01	Llíria	78	38	14	83	11	90	5
18_APIADS02	Bétera	65	5	4	64	9	94	2
18_APIADS03	Llíria	40	ND		ND		ND	
18_APIADS04	Barxeta	77	40	14	94	3	89	6
18_APIADS05	Cheste	75	34	4	54	3	88	3
18_APIADS07	Manises	0	ND		ND		ND	
18_APIADS11	Chiva	51	72	ND	86	ND	97	ND
18_APIADS12	Chiva	76	41	3	80	4	84	2
18_APIADS13	Llíria	59	60	2	72	0	65	7
18_APIADS14	Sagunt	13	71	ND	89	ND	79	ND
18_APIADS15	Monserrat	55	25	6	66	4	68	5
18_APIADS16	Montroy	22	ND		ND		ND	
18_APIADS17	Gestalgar	65	32	2	65	5	63	10
18_APIADS18	Gestalgar	77	19	4	72	5	79	1
18_APIADS19	Yátova	10	ND		ND		ND	
18_APIADS20	Carcaixent	53	49	10	83	7	89	11
18_APIADS21	Guadassuar	0	4	4	59	2	73	6
18_APIADS22	Monserrat	37	37	ND	70	ND	53	ND
18_APICAL01	Beniarrés	65	12	12	80	12	92	9
18_APICAL02	Guardamar del Segura	60	65	9	85	1	81	8
18_APICAL04	Elx	3	68	11	84	4	83	9
18_APICAL05	Guardamar del Segura	62	21	7	89	5	94	3
18_APICAL06	Los Montesinos	68	34	ND	92	ND	81	ND
18_APICAL07	Castalla	26	54	5	76	9	79	6
18_APICAL08	Elx	72	84	7	85	5	82	1
18_APICAL09	Pego	33	55	2	76	6	76	7
18_APICAL10	Pego	8	72	1	74	8	86	ND
18_APICAL11	Beniarrés	53	65	15	92	4	83	2
18_APICAL12	Relleu	10	36	12	55	5	59	10
18_APICAL13	Sant Vicent del Raspeig	62	ND		91	6	92	4
18_APICAL14	Agres	85	45	11	72	6	84	8
18_APICAL15	Real de Montroi	7	34	6	84	1	82	5
18_APIVAL2	Bufali	15	72	8	85	3	80	6
18_APIVAL3	Polinyà del Xúquer	63	37	10	63	2	58	3
18_APIVAL4	Cheste	0	40	8	73	7	63	9
18_APIVAL5	La Font de la Figuera	87	20	8	60	9	53	5
18_APIVAL6	Anna	3	43	ND	86	ND	97	ND
18_APIVAL7	Mariola	89	44	12	87	1	86	6
18_APIVAL8	Bocairent	87	9	3	54	15	69	16
18_APIVAL9	Ontinyent	82	30	13	68	15	79	16
18_CASAPI1	Torás	4	ND		ND		ND	
18_CASAPI3	Nules	0	27	10	76	ND	76	ND

18_CASAPI5	Artana	5	19	6	52	4	63	5
18_PROAPI2	Tous	2	25	3	40	15	75	16
18_PROAPI3	Ayora	2	39	4	60	12	78	6
18_PROAPI5	Ayora	53	23	ND	90	ND	62	ND
18_PROAPI6	Ayora	68	ND		ND		ND	

[1] The expected efficacy of each acaricide was estimated using the mortality values obtained in the bioassays after correction with the mortality in the controls using Schneider–Orelli's formula [1]. [2] Values indicate the percentage of pyrethroid-susceptible mites (SR and SS genotypes). [3] Standard error of the mean. [4] Nondetermined. The mites collected were not enough to conduct the bioassay or only one replicate was carried out.

Table S2. Sample locations and expected efficacy of acaricides recorded in assays from the 2019 season.

Apiary	Location	Expected Efficacy [1] (%)						
		**Pyrethroids [2]**	**Checkmite+**	**SEM [3]**	**Apitraz**	**SEM**	**Apivar**	**SEM**
19_ADSAV01	Llíria	8	ND [4]		ND		ND	
19_ADSAV02	ND	40	98	ND	83	ND	75	ND
19_AIXAM02	Castelló de la Plana	0	71	ND	82	ND	86	ND
19_AIXAM04	Altura	6	ND		ND		ND	
19_ALAPI01	Guardamar del Segura	12	ND		ND		ND	
19_APAC01	Torrechiva	0	ND		ND		ND	
19_APAC02	Vistabella	81	ND		ND		ND	
19_APAC03	Onda	6	54	12	85	4	82	4
19_APAC04	Les Alqueries	10	64	2	72	5	83	7
19_APAC05	Sant Joan de Moró	24	67	6	75	5	76	2
19_APAC06	Borriol	54	73	1	90	3	84	1
19_APAC07	Catí	57	51	2	74	1	74	1
19_APAC08	Xodos	20	48	3	90	0	80	5
19_APAC09	Cervera del Maestre	35	44	ND	87	ND	ND	
19_APAC10	Costur	27	40	13	82	ND	79	ND
19_APAC11	Atzaneta del Maestrat	20	63	5	80	0	80	0
19_APAC12	Ares	8	23	4	80	5	76	5
19_APAC13	Torreblanca	0	24	7	74	4	78	0
19_APIADS01	Montroy	10	72	4	65	14	84[a]	2
19_APIADS02	Casinos	80	38	14	83	2	79	10
19_APIADS03	Montserrat	48	23	ND	ND		ND	
19_APIADS04	Higueruelas	0	ND		ND		ND	
19_APIADS05	Picassent	9	ND		ND		ND	
19_APIADS06	Montroy	72	63	2	77	2	75	4
19_APIADS07	Navarrés	20	21	ND	ND		ND	
19_APIADS08	València	84	54	10	90	7	83	0
19_APIADS09	Llíria	90	23	ND	ND		ND	
19_APIADS10	Chiva	54	50	ND	93	ND	82	ND
19_APIADS11	Alzira	57	63	8	89	2	76	8
19_APIADS12	Llíria	32	37	5	80	4	75	3
19_APIADS13	Buñol	45	62	2	78	4	83	4
19_APIADS14	Torrent	60	82	2	86	1	80	1
19_APIADS15	Chiva	57	53	ND	82	ND	ND	
19_APIADS16	ND	12	ND		ND		ND	
19_APIADS17	Alaquàs	0	69	1	74	1	71	4
19_APIADS19	Villamarxant	74	73	7	76	1	80	ND
19_APIADS20	Cheste	0	61	6	83	2	83	2
19_APIADS21	Cheste	0	60	ND	87	ND	80	ND
19_APIADS22	Chiva	47	52	3	82	0	83	1
19_APIADS23	Chiva	36	48	ND	83	ND	78	ND
19_APIADS24	Bétera	28	60	5	84	1	84	0
19_APIADS25	Bétera	32	55	4	73	1	75	1
19_APIADS26	Bétera	85	30	1	71	3	67	3
19_APIADS27	Chiva	77	56	7	81	4	78	3
19_APICAL01	Molins	18	67	11	85	4	90	3
19_APICAL02	El Ràfol d'Almunia	3	66	2	68	4	74	2
19_APICAL03	Gata de Gorjos	78	33	5	66	6	62	13
19_APICAL04	Benitachell	47	44	ND	89	ND	93	ND

19_APICAL05	La Vila Joiosa	95	52	ND	90	ND	83	ND
19_APICAL06	San Miguel de Salinas	11	40	3	84	5	87	1
19_APICAL07	Torremanzanas	92	46	7	86	1	80	1
19_APICAL08	Torremanzanas	0	59	6	80	3	77	3
19_APICAL09	Finestrat	0	53	1	82	4	82	1
19_APICAL10	Agres	85	69	1	85	3	69	1
19_APICAL11	Castalla	60	81	1	86	1	83	1
19_APICAL12	Sella	55	53	ND	64	ND	69	ND
19_APIVAL01	Quesa	0	60	4	72	4	77	1
19_APIVAL02	La Font de la Figuera	68	36	4	87	2	87	2
19_APIVAL03	Favara	5	31	ND	93	ND	93	ND
19_APIVAL04	Carcaixent	20	52	5	90	0	86	4
19_APIVAL05	Sumacàrcer	0	58	2	86	0	78	2
19_APIVAL06	Bétera	3	29	2	90	2	88	1
19_APIVAL07	Albal	36	57	3	89	1	83	4
19_APIVAL08	Yátova	2	67	4	83	ND	82	ND
19_APIVAL09	Polinyà del Xúquer	47	ND		ND		ND	
19_APIVAL10	Polinyà del Xúquer	40	75	7	76	0	77	1
19_APIVAL11	Bocairent	28	34	3	76	1	77	0
19_APIVAL12	La Vall d'Alcalà	5	68	2	76	5	65	6
19_APIVAL13	Villena	97	40	ND	83	ND	81	ND
19_CASAPI01	Mas de Noguera	49	23	4	79	3	69	4
19_CASAPI03	Tales	17	85	4	94	ND	72	ND
19_CASAPI04	Sacañet	20	75	2	72	2	75	2
19_PROAPI01	Ayora	5	67	ND	85	ND	ND	
19_PROAPI02	L'Énova	0	38	ND	90	ND	83	ND
19_PROAPI03	Orihuela	97	68	1	76	0	72	ND
19_PROAPI04	Requena	89	54	ND	100	ND	72	ND
19_PROAPI05	Moixent	96	40	3	73	3	75	9
19_PROAPI06	Requena	2	85	6	85	2	77	2
19_PROAPI07	Ayora	0	ND		ND		ND	
19_PROAPI08	Ayora	10	83	ND	87	ND	98	ND
19_PROAPI09	Náquera	65	61	1	74	1	89	0
19_PROAPI10	Ayora	72	69	ND	75	ND	ND	
19_PROAPI11	Ayora	50	ND		ND		ND	
19_PROAPI12	Ayora	32	61	10	84	3	79	2

[1] The expected efficacy of each acaricide was estimated using the mortality values obtained in the bioassays after correction with the mortality in the controls using Schneider–Orelli's formula [1]. [2] Values indicate the percentage of pyrethroid-susceptible mites (SR and SS genotypes). [3] Standard error of the mean. [4] Nondetermined. The mites collected were not enough to conduct the bioassay or only one replicate was carried out.

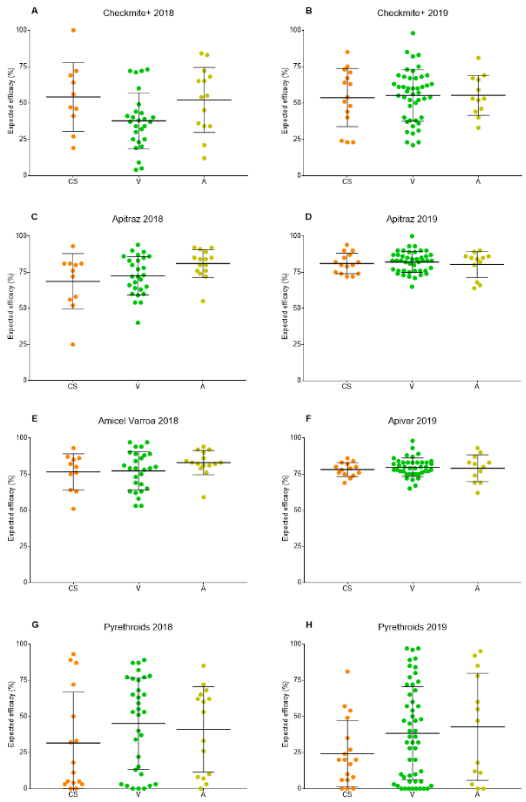

Figure S1. Expected efficacy of acaricides in apiaries providing samples from the three provinces of the Comunitat Valenciana region.

Reference

1. Püntener, W. *Manual for field trials in plant protection second edition*; Ciba-Geigy Limited: Basle, Switzerland, 1981.
